# Progress and Insights Toward an Effective Placental Malaria Vaccine

**DOI:** 10.3389/fimmu.2021.634508

**Published:** 2021-02-25

**Authors:** Benoît Gamain, Arnaud Chêne, Nicola K. Viebig, Nicaise Tuikue Ndam, Morten A. Nielsen

**Affiliations:** ^1^ Université de Paris, Inserm, Biologie Intégrée du Globule Rouge, Paris, France; ^2^ Institut National de la Transfusion Sanguine, Paris, France; ^3^ European Vaccine Initiative, UniversitätsKlinikum Heidelberg, Heidelberg, Germany; ^4^ Université de Paris, MERIT, IRD, Paris, France; ^5^ Centre for Medical Parasitology at Department of Immunology and Microbiology, Faculty of Health and Medical Sciences, University of Copenhagen, Copenhagen, Denmark; ^6^ Department of Infectious Diseases, Rigshospitalet, Copenhagen, Denmark

**Keywords:** *Plasmodium falciparum*, placental malaria, VAR2CSA, PfEMP1, vaccine, pregnancy

## Abstract

In areas where *Plasmodium falciparum* transmission is endemic, clinical immunity against malaria is progressively acquired during childhood and adults are usually protected against the severe clinical consequences of the disease. Nevertheless, pregnant women, notably during their first pregnancies, are susceptible to placental malaria and the associated serious clinical outcomes. Placental malaria is characterized by the massive accumulation of *P. falciparum* infected erythrocytes and monocytes in the placental intervillous spaces leading to maternal anaemia, hypertension, stillbirth and low birth weight due to premature delivery, and foetal growth retardation. Remarkably, the prevalence of placental malaria sharply decreases with successive pregnancies. This protection is associated with the development of antibodies directed towards the surface of *P. falciparum*-infected erythrocytes from placental origin. Placental sequestration is mediated by the interaction between VAR2CSA, a member of the *P. falciparum* erythrocyte membrane protein 1 family expressed on the infected erythrocytes surface, and the placental receptor chondroitin sulfate A. VAR2CSA stands today as the leading candidate for a placental malaria vaccine. We recently reported the safety and immunogenicity of two VAR2CSA-derived placental malaria vaccines (PRIMVAC and PAMVAC), spanning the chondroitin sulfate A-binding region of VAR2CSA, in both malaria-naïve and *P. falciparum*-exposed non-pregnant women in two distinct Phase I clinical trials (ClinicalTrials.gov, NCT02658253 and NCT02647489). This review discusses recent advances in placental malaria vaccine development, with a focus on the recent clinical data, and discusses the next clinical steps to undertake in order to better comprehend vaccine-induced immunity and accelerate vaccine development.

## Introduction

In 2019, approximately 11 million pregnant women were exposed to the risk of *P. falciparum* infection (1). Malaria contracted during pregnancy can lead to significant clinical complications for the mother, including anaemia (2, 3) and hypertension (4, 5), but also for the child. Indeed, malaria in pregnancy may account for over 200,000 stillbirths each year (6) and for the delivery of over 800,000 low birth weight babies (1). This clinical picture varies depending on the parity status and intensity of *P. falciparum* transmission in a given geographical area (7). Primigravid women are highly susceptible to develop severe clinical outcomes following *P. falciparum* infection. However, in high endemicity area, the incidence of illness sharply drops after successive pregnancies (7), demonstrating that protective immunity can be naturally acquired. These observations raised the hope of developing a vaccine that could protect pregnant women against the serious clinical manifestations of malaria in pregnancy.

*P. falciparum* infection in pregnancy can cause substantial morphological and immunological changes in the placenta, where a massive accumulation of infected erythrocytes (IEs) takes place (8–10), reshaping the cytokine profile of the local environment (11–13) and altering the maternofoetal exchanges (14). IEs from placental origin present a unique adhesive phenotype and do not bind to the host receptors (CD36, CD31, EPCR) commonly used by the parasite to cytoadhere to the microvasculature lining (15–17). Instead, placental IEs interact with a low-sulphated sugar only present at the surface of syncytiotrophoblasts, the chondroitin sulphate A (CSA) (18–21). This low-sulphated placental CSA is structurally distinct from CSA present in other organs or secreted into the extracellular matrix and body fluids (21). A single member of the *P. falciparum* Erythrocyte Membrane Protein 1 (PfEMP1) family, named VAR2CSA, has been identified as the main parasite-derived ligand mediating the interaction of IEs with placental CSA (22–27). Numerous studies have correlated the presence of antibodies reacting to VAR2CSA with protection against placental malaria (PM) (28–30). In a recent systematic review and meta-analysis, Cutts et al. showed that VAR2CSA derived antigens were positively associated with the presence of placental infections. However, they could not identify evidence that antibody response towards a specific VAR2CSA antigen is associated with protection from PM (31). The capacity of anti-VAR2CSA antibodies to block the adhesion of IEs to CSA is thought to play a major role in protection (32) but accumulating evidence suggests that other antibody-dependent effector mechanisms, such as opsonic phagocytosis (33–35), could also actively participate in parasite clearance.

Current malaria control strategies during pregnancy mainly consist in the use of long-lasting insecticidal bed nets and the administration of intermittent preventive treatments based on sulphadoxine-pyrimethamine (IPT-SP) as well as iron and folic acid supplementation [reviewed in (36, 37)]. Even if effective in reducing the malaria burden in pregnant women living in endemic areas (1), the global implementation of such approaches is threatened by the emergence of widespread resistance of both anopheles mosquitoes to insecticides (38) and of parasites to anti-malarial drugs (39). Furthermore, since IPT-SP is only initiated at the first antenatal care visit, usually in the second trimester, and that increasing evidence suggests that *P. falciparum* infection is particularly frequent and detrimental in the first trimester of pregnancy (40–43), complementary intervention regimens such as vaccines would therefore be extremely valuable. In the current global malaria vaccine landscape, predominated by anti-infection/transmission approaches, VAR2CSA-based PM vaccines stand as the main anti-disease strategy to reduce placental malaria morbidity and mortality.

## Identifying the Best VAR2CSA-Derived Vaccine Candidate

Prevention of placental infection in first pregnancies is not bestowed by the naturally acquired immune responses from previous *P. falciparum* infections, i.e., prior to pregnancy. Nevertheless, natural and protective immunity develops after one or two pregnancies (7, 44, 45). Vaccine candidates aiming to protect against PM should mimic, at least in part, the naturally acquired antibody response associated with protection (32). Ideally, the vaccine-induced immune responses should be boosted and even substantiated by natural infections.

**Figure 1:**
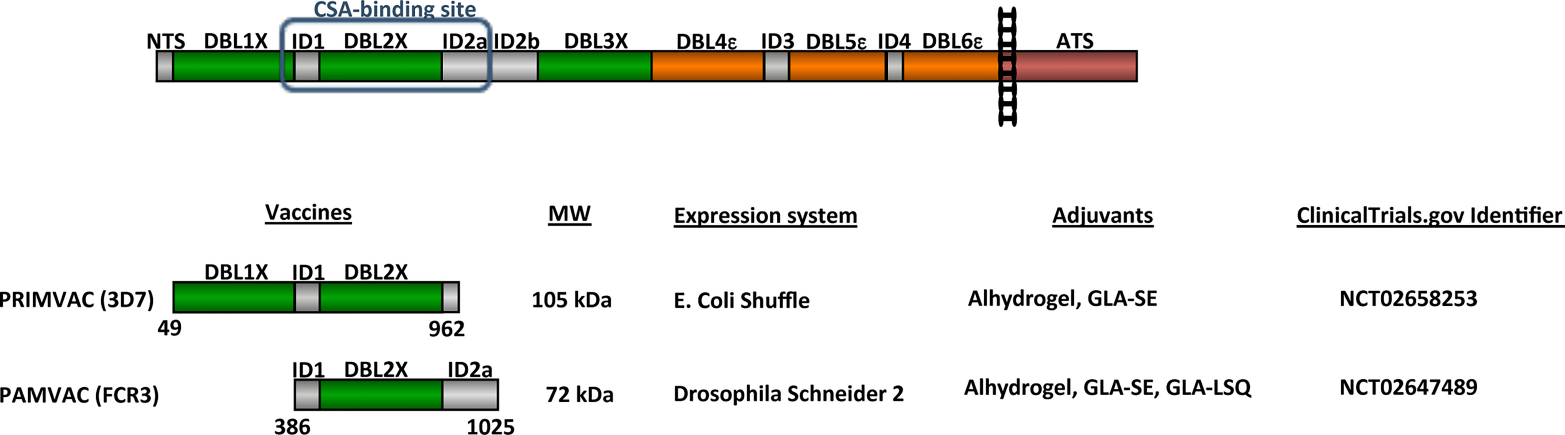
Schematic representation of VAR2CSA and vaccines tested in clinical trials. VAR2CSA is a 350-kDa transmembrane protein composed of an N-terminal segment (NTS) followed by six Duffy-binding like (DBL) domains interspaced by 4 inter-domains (ID), a transmembrane region and a cytoplasmic Acidic C terminus sequence (ATS). PRIMVAC and PAMVAC schematic representation with sequences boundaries, predicted molecular weight, expression systems, and adjuvants used for the Phase I clinical trials. ClinicalTrials identifier for both Phase I are indicated.

Following the identification of VAR2CSA and the determination of its role in PM pathogenesis and immunity, researchers have been trying to identify the best VAR2CSA-derived antigen to include in a PM vaccine (27). Indeed, VAR2CSA is a complex cysteine-rich 350 kDa protein composed of an N-terminal segment (NTS) followed by six Duffy-binding like (DBL) domains interspaced by four inter-domains (ID) with less defined structures, a transmembrane region and a cytoplasmic ATS region (**Figure 1**). This complexity, associated to the difficulty in expressing sufficient material of the full-length VAR2CSA extracellular region to proceed to clinical trial, have spurred researchers to characterize and identify a subunit VAR2CSA vaccine. All *P. falciparum* genomes carry one or several *var2csa* genes (46, 47). *Var2csa* has also been identified in the genomes of *P. reichenowi*, which infects the chimpanzee, indicating that the CSA-binding phenotype has an ancient origin and that protein functionality might exert some degree of constraint on sequence variation (48). Among the various VAR2CSA variants, most of the functional studies have been performed on VAR2CSA derived from 3D7 and FCR3 laboratory strains. The secondary structure of the DBL fold is conserved, and variable regions are scattered in the primary sequence as mosaic blocks, which are generally the prime target of anti-VAR2CSA antibodies (49–51). The assumption that a structurally constrained adhesion-mediating region within VAR2CSA could be more conserved than others, led early research to focus on the determination of the single DBL domain(s) involved in CSA-binding (25, 51–57). The finding that single DBL domains, which appeared not to bind CSA, could induce inhibitory antibodies was a primary indication that the tertiary structure of the protein was not like DBL-pearls on a string (58, 59). When full-length recombinant VAR2CSA proteins became available, low-resolution structures obtained by small-angle X-ray scattering (SAXS) and CSA-binding kinetics of the full-length recombinant proteins indicated that VAR2CSA harboured a globular conformation rather than a linear-shaped structure (60, 61). The determination of the CSA-binding kinetics of the full-length recombinant VAR2CSA also revealed that the full-length protein was able to interact with CSA with high specificity and high affinity, yielding nano-molar affinity constants. This was markedly different to those obtained for single-domain constructs in the micromolar range (60, 61). Using SAXS and single particle reconstruction from negative stained electron micrographs, a recent study confirmed the globular conformation of VAR2CSA and suggested a model for CSA-binding (62). Dissection of the CSA-binding affinities of recombinant truncations of full-length VAR2CSA, as opposed to single DBL domains, identified the N-terminal part of VAR2CSA as the CSA-binding region (63–65). While for the 3D7 variant, a more specific binding to CSA was observed using the DBL1x-DBL3x multidomain protein (64), the DBL1x and DBL3x domains did not appear as essential for high affinity CSA binding for the FCR3 variant (65). The binding affinity of FCR3 DBL2x including small N and C terminal flanking ID1 and ID2 inter-domains was similar to the autologous full-length VAR2CSA. Thus, the minimal binding region of FCR3-VAR2CSA was designated as ID1-DBL2x-ID2a (65).

Although the identification of the high affinity CSA-binding site has orientated researchers to target this region to induce inhibitory antibodies, many studies have looked at the quality of the immune response generated by the different single DBL domains and multiple domains, including the full-length extracellular region of VAR2CSA. Indeed, due to the globular fold of the full-length molecule it appears possible that antibodies directed towards residues not directly involved in CSA-binding could i) be cross-reactive against different variants and/or ii) affect the molecular interaction of VAR2CSA with CSA by steric hindrance.

Interestingly, the generation of single domain nanobodies following immunization of camelids with the full-length VAR2CSA recombinant protein revealed a preferential targeting of the DBL1x domain and, to a lower extent, of DBL4ϵ, DBL5ϵ, and DBL6ϵ (66) suggesting the existence of immunodominant epitopes outside the CSA-binding region of the protein. This assumption is also strengthened by another study showing that monoclonal antibodies generated from naturally infected women are predominantly directed against DBL3x and DBL5x (67). Remarkably, none of these nanobodies and monoclonal antibodies presented CSA-binding inhibitory capability, which indicates that a single molecule targeting an epitope outside the binding region is unlikely to inhibit the adhesion of IEs to CSA.

Several small animal immunization studies, many of which have been carried out in collaboration between different laboratories, have established that the generation of antibodies targeting epitopes shared between different parasite lines could be achieved using various VAR2CSA domains (68–75). Although antibodies raised against the full-length VAR2CSA may not be cross-inhibitory (76), several studies performed in rodents have shown that shorter constructs including the N-terminal region of VAR2CSA are able to induce cross-inhibitory antibodies preventing IEs from binding to CSA (77–81).

Taken together, these data have evolved the rationale for developing a vaccine against PM based on the CSA binding N-terminal region, in the hope that it will prime the immune response towards semi-conserved antigenic determinants and then mimic the antibody repertoire acquired through natural exposure in first pregnancy (32).

## PRIMVAC and PAMVAC Phase I Clinical Trials

VAR2CSA-based vaccines spanning the CSA-binding region (PRIMVAC and PAMVAC), designed to generate antibodies capable of inhibiting IEs adhesion to placental cells, have recently been assessed in two separate phase I clinical trials (ClinicalTrials.gov identifiers NCT02658253 and NCT02647489, respectively) (**Figure 1**). Together they represent an attractive intervention strategy to protect primigravid women from the serious clinical outcomes of PM. PAMVAC expressed in a Drosophila Schneider 2-derived cell line spans the interdomain region 1 through Duffy binding-like domain 2 to inter-domains 2 (ID1-DBL2x-ID2a) of the FCR3 variant of VAR2CSA (82), while PRIMVAC expressed in *E. coli* SHuffle^®^ is based on the DBL1x-2x multidomain of VAR2CSA from the *P. falciparum* 3D7 strain (**Figure 1**) (79, 80, 83). In order to allow comparative assessment of different placental malaria vaccine candidates, especially PRIMVAC and PAMVAC, two workshops hosted by the European Vaccine Initiative allowed the harmonization of clinical development plans and of assays for immunological assessment (84).

Based on these recommendations, both clinical trials were designed as randomized, double-blind, placebo-controlled, dose escalation trials and were meant to evaluate the safety and immunogenicity of three intramuscular vaccinations with progressively higher doses. PRIMVAC was adjuvanted with Alhydrogel or glucopyranosyl lipid A adjuvant in stable emulsion (GLA-SE) whereas PAMVAC was adjuvanted with Alhydrogel or GLA-SE or injected as a liposomal formulation in combination with QS21 (GLA-LSQ) (**Figure 1**). Volunteers were recruited into sequential cohorts receiving 20 μg, 50 μg, or 100 μg of vaccines at day 0, 28, and 56. PAMVAC volunteers were observed for 6 months following last immunization, while PRIMVAC volunteers were followed for one year after the last vaccination. While the safety and immunogenicity of PRIMVAC has been recently published in both malaria naïve (French volunteers) and *P. falciparum*-exposed non-pregnant women (Burkinabe women) (83), only the clinical evaluation of PAMVAC in malaria naïve volunteers (German volunteers) has been so far reported (82). All PAMVAC and PRIMVAC formulations were safe and well tolerated and none of the vaccines induced serious adverse events (82, 83).

PAMVAC and PRIMVAC were immunogenic in all participants and antibody levels were usually higher for both vaccines when adjuvanted with GLA-SE as compared to Alhydrogel and GLA-LSQ, at all dosages. In the case of PRIMVAC, all the volunteers seroconverted after two vaccine doses and a high proportion of them were still seroconverted one year after the third vaccination, which is indicative of a long-lasting immunity. Interestingly, IgG subclass analysis revealed that the induced antibodies were mostly IgG1 and IgG3 for PRIMVAC vaccinated volunteers. Therefore, the PRIMVAC-induced antibody response seems to mimic the naturally acquired immune response observed in multigravid women (85, 86).

Both vaccines generated antibodies reacting with the homologous VAR2CSA expressing parasites (NF54 for PRIMVAC and FCR3 for PAMVAC), however limited cross-reactivity was observed against heterologous VAR2CSA variants either recombinantly expressed (PAMVAC trial) or expressed on the surface of FCR3-CSA and 7G8-CSA IEs (PRIMVAC trial). The highest cross-reactivity was observed in sera collected from women that received the 100 μg PRIMVAC dose. Both vaccines induced functionally active antibodies inhibiting the interaction of their corresponding homologous VAR2CSA expressing IEs to CSA but low or no CSA-binding inhibition was observed for IEs expressing heterologous VAR2CSA variants (82, 83).

## Discrepancies Between Preclinical and Clinical Studies

Interestingly, the PAMVAC and PRIMVAC vaccines were able to induce antibodies in humans at significantly high titers after three doses received by the study participants. These antibodies were mainly vaccine-specific and mainly possessed functional activity against the homologous strains. Although these antibodies made it possible to describe a cross-reactivity with the constructs originating from other parasite variants, in particular those induced by vaccines adjuvanted with GLA-SE (82, 83), the lower cross-reactivity and the lack of cross-inhibition contrasts with the data generated in small animal models that guided the selection of these vaccine candidates (63, 77, 79, 80).

The observation that the responses in humans appear less cross-reactive than in rodents clearly raises the question of the transferability of the data generated in small animal models to humans. One confounding effect could be that the rodents received a higher dose per bodyweight than humans, so that the induced antibody titers are overall lower in humans compared to small animal models. A lower antibody titer would then possess less cross-reactive antibodies and then explain the lowest observed cross-reactivity. It is also possible that there is species-specific selection for cross-reactive epitopes in rodents versus humans. This would be a major obstacle in the placental malaria vaccine development process where the lack of an appropriate animal model remains. An animal model that would be as close as possible to humans, like non-human primates could be an asset in the preclinical validation phases. Efforts to develop such a model in the Aotus monkey is currently underway and could offer an interesting track in this perspective.

Interestingly, it was noted that some nulligravid women participating in the study in Burkina Faso to receive the PRIMVAC vaccine, possessed VAR2CSA antibodies before the administration of the vaccine doses (83). While these observations corroborate those of a few studies which have shown the presence of antibodies reacting with certain VAR2CSA antigens in some non-pregnant or male subjects living in endemic regions (87–89), their potential interference or benefit with vaccination deserves to be considered.

## Current Vaccine Development Gaps and Potential Future Developments

The preclinical and phase I clinical trial results for PAMVAC and PRIMVAC are highly encouraging and confirm the feasibility of developing a PM vaccine through further clinical testing. However, prior to embarking on costly, large-scale phase II clinical trials, it is essential to further evaluate the longevity of the immune response in vaccinated women and meanwhile assess its development upon natural infections. Novel strategies to enhance the immunogenicity of the PM vaccine candidates and to maximize the antibody cross-reactivity against different VAR2CSA variants could also be envisaged.

### Longevity

Longevity of the vaccine-induced immune response is an essential element in placental malaria vaccine development, since nulligravid women will be vaccinated many months or years prior to their first pregnancy. In 2012, Fowkes et al. suggested that half-lives of antibody responses in pregnant women induced by natural *P. falciparum* infection were in general relatively short for merozoite antigens (0.8–7.6 years) (90). However, half-lives of antibodies to VAR2CSA were significantly longer (36–157 years), suggesting that antibodies acquired in one pregnancy may be maintained to protect subsequent pregnancies (90). This raises hope for PM vaccine development, a hope that is further bolstered by the results obtained in the pre-clinical and clinical PM vaccination studies. The PM vaccine candidates, especially PRIMVAC, produced a long-lasting immune response in PRIMVAC vaccinated women who developed an immune response that lasted for more than a year (83). To further characterize the longevity of the PRIMVAC-induced immune response in women in malaria-endemic areas and to define vaccination age and frequency of booster vaccinations, follow-up of the vaccinated women for several years will be crucial. Additionally, the capacity of the vaccine candidates to boost and broaden a naturally acquired immune response would provide valuable insight.

### Immunogenicity

Recombinant soluble proteins are often thought to induce an immune response of insufficient strength and breadth to confer full protection. Therefore, alternative approaches for the presentation of the VAR2CSA antigens are considered. The most advanced approach is using a capsid-like particle (CLP) that has been added as a backbone to the PM antigen, thereby possibly inducing a stronger immune response than a vaccine based on soluble recombinant protein and thus potentially improving immunogenicity, cross-reactivity and longevity of the induced immune response (91, 92).

### Cross-Reactivity

Each vaccine candidate, PAMVAC and PRIMVAC, consists of a single recombinant protein. However, VAR2CSA is a diverse antigen and in Benin alone, 57 haplotypes of the vaccine target were identified which phylogenetically cluster into five clades (93). The PM vaccine candidates PRIMVAC and PAMVAC are from two distinct clades; 3D7 (clade 1) and FCR3 (clade 2) respectively. Interestingly, the authors found an association between the 3D7-like clade and low birthweight (93). Because of the extensive VAR2CSA polymorphism, the development of a PM vaccine is challenging. While the correct part of VAR2CSA to target appears established, it may be needed to generate second-generation vaccines, which expands the possibility of immunizing against several variants to improve cross-reactivity.

Different approaches may be evaluated: 1) prime - boost vaccination using VAR2CSA variants from different clades, 2) combination vaccination approaches either using, e.g., soluble recombinant proteins or multi-VAR2CSA-CLPs, 3) chimeric VAR2CSA variants, either based on the PAMVAC or PRIMVAC protein boundaries or rational structure-based designs. Finally, the recently hyped mRNA vaccine technology deployed against Covid-19 may be a way to incorporate several variants due to the ease and low cost of manufacture (94).

## Conclusion

Over the past years, a promising portfolio of PM vaccine candidates was developed and two VAR2CSA-derived vaccines were brought to clinical evaluation, confirming the feasibility of developing a PM vaccine. However, to evaluate and compare the results of the various PM vaccine approaches, the harmonization of clinical trial procedures and the standardization of immunoassays that was initiated before the start of the phase I clinical trials (84) has to be further strengthened. Stringent go/no-go criteria will be required to decide to enter in the next development stages based on production feasibility, safety, and induction of cross-reactive and functional immune responses. A major obstacle to transitioning a PM vaccine from preclinic to clinic is the lack of a suitable animal model (95). Evaluation of PM vaccine efficacy is particularly complex. Indeed, the lack of surrogates to predict PM vaccine-induced protection limits the potential of early clinical trials to provide indications on vaccine efficacy. The amplitude of the antibody response resulting from vaccination appears today as an indicator of putative efficacy, although the threshold levels of antibodies required for protection are undetermined. The lowest cross-reactivity observed during the clinical trials in comparison to the preclinical evaluation could be the consequence of a lower amplitude of the immune response upon antigen exposure in humans compared to rodents. Since sterile immunity is not required for a PM vaccine, the hope is that the vaccine-induced response will be boosted and even broadened by natural infection. Alternate schedules of immunization, antigen dosage, and combinations with other VAR2CSA-based vaccines are under development and will be assessed in future studies for their capacities to broaden the cross-reactivity of the induced immunity against heterologous VAR2CSA variants and then fully protect women from the negative outcomes of PM.

## Author Contributions

BG, AC, NV, NTN, and MN wrote the manuscript. All authors contributed to the article and approved the submitted version.

## Funding

This work is supported by the French National Research Agency (ANR-16-CE11-0014-01), grants from Laboratory of Excellence GR-Ex, reference ANR-11-LABX-0051 and the French Parasitology consortium ParaFrap (ANR-11-LABX0024). The labex GR-Ex is funded by the program “Investissements d’avenir” of the French National Research Agency, reference ANR-11-IDEX-0005-02. Funding for PRIMVAC and PAMVAC activities were provided by the Bundesministerium für Bildung und Forschung, through Kreditanstalt für Wiederaufbau (ref: 202060457), Germany; Inserm and Institut National de Transfusion Sanguine, France; Irish Aid, Department of Foreign Affairs and Trade, Ireland. European Union in the Seventh Framework Programme (FP7-HEALTH-2012-INNOVATION; under grant agreement 304815), the Danish Advanced Technology Foundation (under grant number 005-2011-1).

## Conflict of Interest

MN appears on a patent issued on virus like particle vaccines (US10086056B2).

The authors declare that the research was conducted in the absence of any commercial or financial relationships that could be construed as a potential conflict of interest.

The handling editor declared a past collaboration with the author NTN.
